# CB-LPD, MGUS, T-LGLL, and PRCA: A rare case report of 4 concomitant hematological disorders

**DOI:** 10.1097/MD.0000000000027874

**Published:** 2021-11-24

**Authors:** Qinhong Xu, Jieni Yu, Xiaoyan Lin, Youli Li, Kejie Zhang

**Affiliations:** a Department of Hematology, Zhongshan Hospital, Xiamen University, Fujian Medical University Clinic Teaching Hospital, Xiamen, Fujian, China; b The Center of Clinical Laboratory, Zhongshan Hospital, Xiamen University, Fujian Medical University Clinic Teaching Hospital, Xiamen, Fujian, China.

**Keywords:** case report, clonal B-cell lymphoproliferative disorders, monoclonal gammopathy of undetermined significance, pure red cell aplasia, T large granular lymphocyte leukemia

## Abstract

**Rationale:**

Monoclonal gammopathy of undetermined significance (MGUS) is a clinically asymptomatic clonal plasma cell or lymphoplasmacytic proliferative disorder. Recently, some case reports have described the association of pure red cell aplasia (PRCA) with MGUS, even with a relatively low monoclonal immunoglobulin burden. T large granular lymphocyte leukemia (T-LGLL) is a chronic lymphoproliferative disorder characterized by clonal expansion of T large granular lymphocytes, which is rare in China. There are some reports about T-LGL leukemia in patients with B-cell lymphoma; however, it is very rare that T-LGLL coexists with MGUS and clonal B-cell lymphoproliferative disorders (CB-LPD).

**Patient concerns:**

A 77-year-old man was hospitalized because of anemia. He was diagnosed with MGUS, CB-LPD, and PRCA. During the development of the disease, a group of abnormal T lymphocytes was detected by flow cytometry of peripheral blood.

**Diagnosis:**

Combining clinical manifestations with the result of T cell receptor gene rearrangement and immunophenotype, it was consistent with the diagnosis of T large granular lymphocyte leukemia.

**Interventions:**

The patient was treat with bortezomib and dexamethasone regimen, Rituximab and sirolimus.

**Outcomes:**

The patient was transfusion independent after therapies.

**Lessons:**

We report a patient with 4 concomitant hematological disorders: T-LGLL, MGUS, CB-LPD, and PRCA, aiming to represent the clinical and flow cytometry characteristics of these concomitant diseases, analyze the mechanism between diseases, and provide a clinical reference.

## 1 Introduction

The main characteristic of bone marrow in patients with pure red cell aplasia (PRCA) is erythrocyte hypoplasia and dysmaturation, whereas other marrow elements are normal.^[1]^ Some reports have indicated that the destruction of erythroid precursors is related to humoral or cellular immunity. The etiology of idiopathic PRCA remains unclear, and immunosuppressors are the basic treatments.^[2]^ While, Acquired PRCA is commonly associated with thymoma, lymphoproliferative disorders, viral infection, and autoimmune diseases.^[3]^

The control of secondary causes is closely associated with the prognosis of acquired PRCA. The most commonly reported lymphoproliferative diseases with PRCA are T large granular lymphocyte leukemia (T-LGLL) and chronic lymphocytic leukemia (CLL), and other types of lymphoma, myeloma, and Waldenstrom's macroglobulinemia have also been reported.^[4,5]^ Although the co-existence of T-LGLL and clonal B-cell lymphoproliferative disorders (CB-LPD) can be found in the literature,^[6]^ it is extremely rare that of 3 simultaneous diseases of T-LGLL and CB-LPD with monoclonal gammopathy of undetermined significance (MGUS) in the same patient. Here, we report a patient with T-LGLL, CB-LPD, and MGUS with clinical presentation of PRCA, which has not been reported so far.

## 2 Case report

A 77-year-old man was hospitalized on April 15, 2019, because of dizziness and fatigue for half a month. No significant weight loss is observed. He had a history of chronic kidney disease, hypertension, and degenerative diseases of the lumbar spine. There were no positive signs except for an anemic appearance. The results of laboratory tests were as follows (Table 1): moderate to severe large cell anemia, significantly reduced reticulocyte count, and erythrocyte rouleau formation. Disordered iron metabolism manifested as an increase in serum ferritin and serum iron, rather than total iron binding capacity. Immunoglobulin quantification revealed high immunoglobulin G antibody (IgG) levels. The serum erythropoietin level was greater than 747 mIU/ml. The serum-free light chain showed a high level of kappa and lambda, while the ratio was normal.

**Table 1:** The result of laboratory tests.

Item	Database	NR	Item	Database	NR
WBC	5.94 × 10^9^/l	3.5–9.5 (×10^9^/l)	Serum iron	51.2 μmol/l	10.6-36.7 μmol/l
ANC	3.31 × 10^9^/l	1.8–6.3 (×10^9^/l)	Serum ferritin	643.5 ng/ml	30.0-400.0 ng/ml
PLT	376 × 10^9^/l	125–350 (×10^9^/l)	TIBC	57.8 μmol/l	50.0-77.0 μmol/l
RBC	1.32 × 10^12^/l	4.3–5.8 (×10^12^/l)	EPO	>747 mIU/ml	2.59-18.5 mIU/ml
RET	0.004 × 10^12^ g/L (0.28%)	0.024–0.084 (×10^12^/l) (0.50%–1.50%)	IgG	16.1 g/l	7.51-15.60 g/l
Hb	47 g/l	130–175 g/l	IgA	1.91 g/l	0.8-4.53 g/l
MCV	105.3 fl	82–100 fl	IgM	1.36 g/l	0.46-3.54 g/l
MCH	35.6 pg	27–34 pg	SFLC κ	47.4 mg/l	1.7-3.7 mg/l
HCT	18.5%	40%–50%	SFLC λ	37.9 mg/l	0.9-2.1 mg/l
Creatinine	106.4 μmol/l	57.0–111.0 μmol/l	κ/λ	1.251	1.35-2.65

ANC = absolute neutrophil count, EPO = erythropoietin, Hb = hemoglobin, HCT = hematocrit, IgA = immunoglobulin A antibody, IgG = immunoglobulin G antibody, IgM = immunoglobulin M antibody, MCV = Mean corpuscular volume, MCH = Mean corpuscular hemoglobin, NR = normal range, PLT = platelet, RBC = red blood cell, RET = reticulocyte, SFLC κ = serum-free light Kappa chain, SFLC λ = serum-free light Lambda chain, TIBC = total iron binding capacity, WBC = white blood cell.

Bone marrow karyotype:46, XY [3]/45, X,-Y[2]. The bone marrow MYD88 gene mutation detected by reverse transcription PCR was negative.

**Figure 1:**
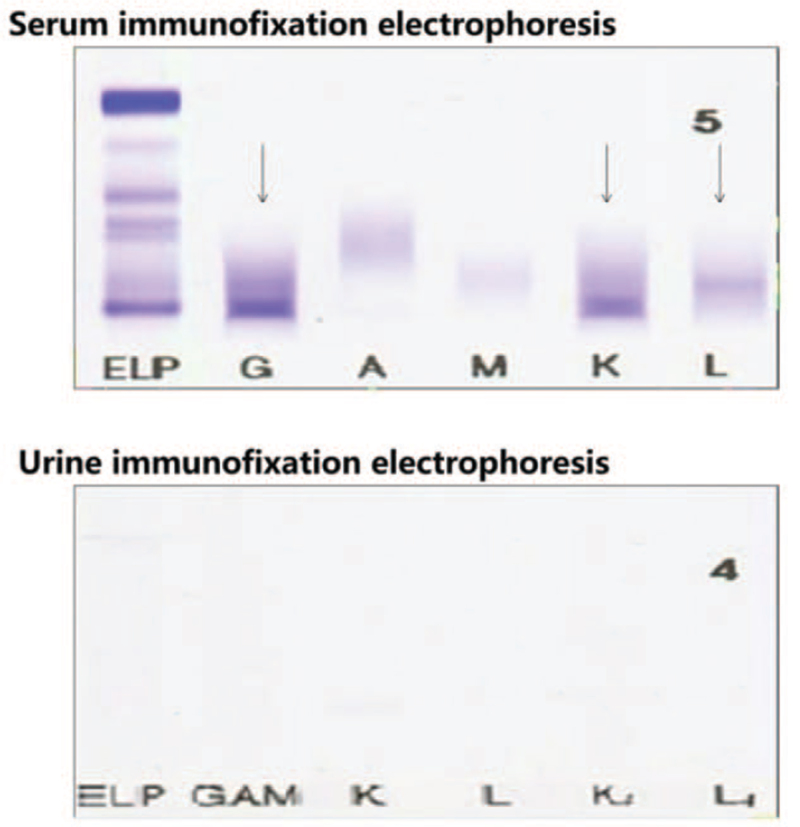
Serum immunofixation electrophoresis: there are 2 M protein bands on ELP, 1 of which forms a specific reaction precipitation band with anti-IgG and anti-Kappa, and the other forms a specific reaction precipitation band with anti-Lambda: Urine immunofixation electrophoresis: there was no M protein band on ELP. ELP = elastin like protein.

**Figure 2:**
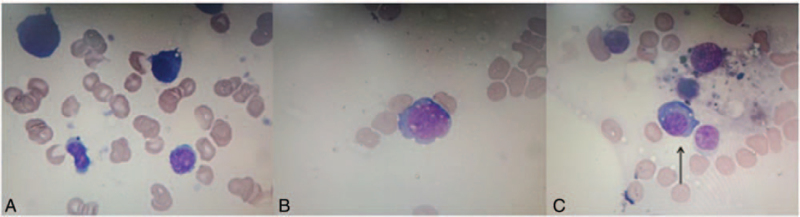
Photomicrograph of first bone marrow aspirate (Wright's-Giemsa X1000): absence of immature erythrocytes (A), unclassified cells (B), hemophilic phenomenon (C).

**Figure 3:**
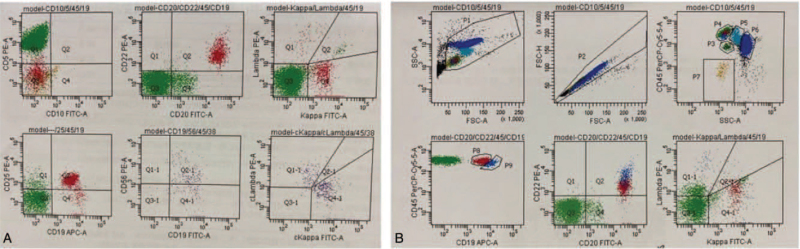
Representative example of 4-flow cytometry study of CB-LPD case. Abnormal mature B lymphocytes (show in red and emphasized) are identified by their expression of CD19(dim), CD20, CD22(dim) and monoclonal expression of surface Kappa light chain, absence of expression of CD5, CD10 and surface lambda light chain. All remaining viable lymphocytes are in green and normal mature B lymphocytes are in blue. CB-LPD = clonal B-cell lymphoproliferative disorders.

Serum protein electrophoresis indicated 2 M protein bands (IgG-KAP+LAM) (Fig. 1). We observed many unclassified cells in the bone marrow morphology (Fig. 2). Flow cytometry revealed a group of abnormally mature B lymphocytes (Fig. 3). No significant abnormalities were observed in bone marrow biopsy pathology.

Imaging examination: There was no Lymphadenopathy or bone destruction throughout the body.

**Figure 4:**
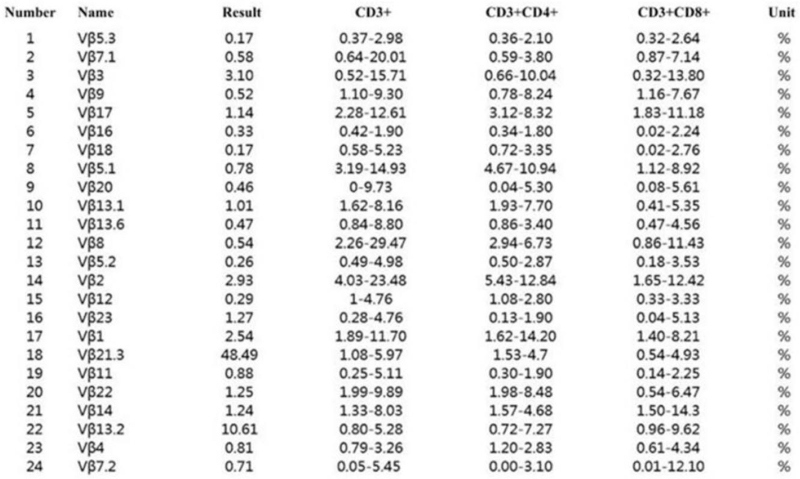
TCRVβ flow cytometric analysis of peripheral blood: CD3^+^CD4^+^ T lymphocytes are normal; While the proportion of TCR Vβ21.3 subfamily of CD3^+^CD8^+^T lymphocytes was increased (48.49%). T cell clonal diseases cannot be excluded. TCR = T cell receptor.

Finally, the diagnosis was as follows: 1. MGUS (IgG-KAP+LAM); 2. PRCA; 3. CB-LPD. The bortezomib and dexamethasone regimen (bortezomib 1.9 mg d1, d8, d15, d22, dexamethasone 10 mg d1-2, 8-9, 15-16, 22-23) was administered on May 7, 2020, for 1 week. The patient's fatigue symptoms improved and he was independent of transfusion; and his hemoglobin level reached 70 g/l after 1 week of therapy. He refused to continue therapy. Repeated routine blood examination showed a gradual increase in hemoglobin levels, even up to 119 g/l. However, after 5 months of discontinuation of treatment (October, 2019), the hemoglobin level gradually decreased to 74 g/l. The patient visited the hospital again. Immunoglobulin, serum protein electrophoresis, immunofixation electrophoresis, free light chain, bone marrow cytology, bone marrow pathology, and bone marrow flow cytometry examination were similar to the previous results. However, in addition to abnormal monoclonal B lymphocytes, a group of abnormal T lymphocytes was detected by flow cytometry of peripheral blood (Fig. 4), as follows: CD4^+^T lymphocytes were normal; and the proportion of T cell receptor (TCR) Vβ21.3 subfamily of CD8^+^T lymphocytes was 48.49%. A clonal T-cell population with TCRr row flow IgH gene rearrangement and an abnormal immunophenotype was identified; According to T-LGLL principle of treatment, cyclosporine 100 mg twice a day and prednisone 40 mg/d and thalidomide 50 mg/d, methotrexate 25 mg/wk + cyclophospamide 100 mg/d were given sequentially. The patient was still transfusion-dependent. Rituximab 700 mg treatment on July 11, 2020, July 18, 2020, and July 25, 2020, respectively. The hemoglobin level reached 85 g/l on July 31, 2020. The patient felt less weak than before. However, after 2 months, the hemoglobin level still decreased, and the patient was transfusion dependence. Accordingly, the patient was administered sirolimus orally (2 mg/d) on January 2021, and his hemoglobin level continued to rise to 120 g/l for 6 months.

## 3 Discussion

Recently, there have been some published case reports describing the simultaneous presence of PRCA with MGUS, T-LGLL, and CB-LPD. ^[5,7,8]^ And, multiple mechanisms between PRCA and T-LGLL have been postulated. The inhibition of erythropoiesis by T-LGL and chronic antigenic stimulation may play an important role.^[9]^ Secondary PRCA in the setting of CLL is associated with polyclonal high-affinity IgG, which is directed against maturing erythroblasts in the bone marrow.^[10]^ As for CB-LPD-related PRCA, monoclonal proteins can directly inhibit the differentiation of bone marrow burst forming unit-erythroid colonies. ^[11]^ What's more, monoclonal protein mediated inhibition of erythrocyte maturation seem to be independent on tumor burden, as this phenomenon has been observed both with high and low levels of monoclonal protein (such as MGUS).^[12]^

T-LGL leukemia is an indolent disease. A substantial proportion of patients with T-LGL leukemia have hematological disorders, including lymphoid and nonlymphoid lineages. The spectrum of clonal B-cell neoplasms in T-LGL leukemia includes CLL and non-Hodgkin lymphoma. T-LGLL can precede, follow, or present synchronously with B-cell dyscrasias.^[13]^ Anemia and neutropenia are the most common manifestations, whereas autoimmune diseases, such as rheumatoid arthritis, are less common.^[14]^ Recently, some cases of T-LGLL with MGUS have been reported.^[15]^ About 52% of the patients were female. Major hematologic complications include anemia and neutropenia, whereas splenomegaly is relatively rare. Half of the patients have IgG monoclonal protein, and some MGUS patients progress to multiple myeloma or lymphoplasmacytic lymphoma (LPL). However, to the best of our knowledge, the concomitant presence of T-LGLL, MGUS, and CB-LPD is extremely rare. Only 1 case has been reported in the literature.^[16]^ In this case, an 88-year-old man presented with weakness and no hematologic complications, lymphadenopathy, or hepatosplenomegaly. CD4^+^/CD8 weak T-LGL cells can be found in both peripheral blood and bone marrow, and the clonality of T-LGLL was confirmed by polymerase chain reaction. In addition, 2 different B-cell clones can be found in flow cytometry analysis, given that plasma cells of MGUS are a cytoplasmic kappa light-chain restriction population, while B-lymphocytes of monoclonal B-cell lymphocytosis (MBL) exhibited a surface lambda light-chain restriction population. This finding suggests that T-LGL cell expansion may be a direct anti-tumor immunological response to both B-cell and plasma-cell aberrant populations. The patient was admitted to the hospital because of anemia. Subsequently, a diagnosis of PRCA was established based on bone marrow biopsy. He had no hepatosplenomegaly or myeloma-related organ damage. Abnormal proliferation of monoclonal B lymphocytes was observed in both bone marrow and peripheral blood. Serum immunofixation electrophoresis revealed IgG-KAP+LAM. Treatment of plasma cells and abnormal B lymphocytes could improve the symptoms of anemia, and the results of flow cytometry analysis were consistent with the immunophenotypic characteristics of LPL. Therefore, it makes sense to consider LPL. However, plasma cells exhibited polyclonal expression and the MYD88 mutation was negative in the bone marrow. The insensitivity of flow cytometry and PCR gene detection methods may be a factor. During the development of the disease, the patient had a large granule T lymphocyte proliferation in the peripheral blood. The diagnosis of T-LGLL was established by combining TCR gene rearrangement results. The case presented in our report with 4 hematological disorders is rare and has not been reported in the literature to date.

Therapy for acquired PRCA varies with different pathogenies. Rituximab seems to be a highly effective and safe selection for PRCA that complicates CB-LDP.^[17,18]^ As for PRCA with LGL leukemia, some immunosuppressive therapy have been applied with variable success, such as methotrexate, cyclosporine, cyclophosphamide, prednisone, hematopoietic growth factors, and nucleoside analogs.^[19]^ Recent publications suggest that small clone monoclonal immunoglobulinemia can produce severe clinical manifestations by involving many organs.^[20–22]^ To better characterize these situations, monoclonal gammopathy of clinical significance (MGCS) was proposed.^[23]^ In clinical work, we often ignore MGCS, so we should pay more attention to it. However, research on MGCS has never stopped. Treatments based on rituximab, lenalidomide, and bortezomib for monoclonal protein-related diseases have been recommended.^[24,25]^ As for our patient, his anemia has been improved after the bortezomib and dexamethasone protocol as well as rituximab treatment. However, when T large-granule lymphocytes emerged, treatment with T-LGLL was ineffective. This suggests that PRCA may be associated with M-protein produced by monoclonal B lymphocytes. Some studies suggest that T-LGLL in the course of disease may be a direct immune surveillance response to aberrant B cell populations or abnormal T cell proliferation caused by foreign antigens in the condition of long-term B cell dysfunction. ^[26,27]^ In recent years, some new findings regarding the mechanism of T-LGLL with CB-LPD have been reported. Studies have found that BTK inhibitors can induce the expansion of CD57^+^ T large granular lymphocytes (LGLs) in Philadelphia chromosome-positive malignancies. In addition, patients with LGLs showed stronger molecular responses. ^[28–30]^

Generally, patients with indolent T-LGLL usually have a favorable prognosis, with a median overall survival of more than 10 years. They need no treatment until the development of recurrent infection, severe neutropenia, symptomatic anemia, etc. ^[31]^ T-LGLL and B cell disease often coexist. ^[32,33]^ For T-LGLL with MGUS, studies have reported an indolent course of disease in approximately 46% of patients. Only 15% of patients were in remission, and 38% of patients relapsed after immunosuppression and targeted therapy.^[14]^ While, For T-LGLL with other clonal B-cell lymphoproliferative disorders, the death rate seemed to be higher.^[34]^ Some PRCA cases are related to T-LGLL. Patients always present with symptoms of anemia and are transfusion dependent. Immunosuppressive therapy resulted in a median survival of 60 months.^[35]^ However, when complications occur, the overall survival is significantly reduced. Tanaka et al^[36]^ reported a T-LGLL patient combined with PRCA who developed EBV-associated hemophagocytic syndrome after immunosuppressive therapy; the disease rapidly progressed and the patient died 1 week later. As for PRCA with lymphoproliferative diseases (such as CLL), the majority of patients could achieve complete remission after rituximab treatment, with a mean duration of remission of 18.5 months and a maximum duration of 60 months.^[37,38]^ Additionally, the effective treatment of MGUS with PRCA is unclear. A few case reports have shown that treatment with bortezomib and dexamethasone resulted in a rapid reduction of M protein and a treatment-free survival of 8 months.^[39]^

To better understand the pathophysiology of these disorders, it is necessary to investigate the intriguing association of T-LGLL with MGUS or CB-LPD. Given the extreme rarity of 4 simultaneous diseases in the same patient, case reports touching clinical features, immunophenotypic, and molecular characteristics are useful to provide more information about this unusual assembly of hematological disorders and the possible mechanisms involved in its appearance.

In conclusion, our case indicates that T-LGL leukemia can coexist with B cell dysfunction and/or B cell lymphoproliferative diseases, including MGUS, and the exact pathophysiologic mechanism awaits further clarification. Furthermore, it is important to emphasize the value of dynamic peripheral blood flow cytometry and immunofixation electrophoresis, which may pass unnoticed on bone marrow morphology and flow cytometry examination.

## Acknowledgments

The authors appreciate Qinqin Li for revising our manuscript.

## Author contributions

**Conceptualization:** Kejie Zhang.

**Data curation:** Qinhong Xu, Jieni Yu, Youli Li.

**Funding acquisition:** Yongli Zhang, Kejie Zhang.

**Visualization:** Xiaoyan Lin.

**Writing – original draft:** Qinhong Xu.

**Writing – review & editing:** Kejie Zhang.
